# Statement of Retraction: Next-generation -omics approaches to drive carboxylate production by acidogenic fermentation of food waste: a review

**DOI:** 10.1080/21655979.2026.2641946

**Published:** 2026-03-12

We, the Editors and Publisher of the journal *Bioengineered*, have retracted the following article from publication.

Kumar, R., Kumar, R., Brar, S. K., & Kaur, G. (2022). Next-generation -omics approaches to drive carboxylate production by acidogenic fermentation of food waste: a review. *Bioengineered*, 13(7–12), 14987–15002. https://doi.org/10.1080/21655979.2023.2180583

Following publication, internal integrity audits revealed concerns around the stringency of the peer review and editorial decision-making process of this article.

Following an investigation by the Taylor & Francis Publishing Ethics & Integrity team in full cooperation with the current Editor, it was confirmed that this article was not peer reviewed in line with the Journal’s peer review standards and policy.

As the stringency of the peer review process is core to the integrity of the publication process, the Editor and Publisher have decided to retract the article. The journal has not confirmed if the authors were aware of this insufficient peer review process.

The journal is committed to correcting the scientific record and will fully cooperate with any institutional investigations into this matter. The authors have been informed of this decision.

We have been informed in our decision-making by our policy on publishing ethics and integrity and COPE guidelines.

The retracted article will remain online to maintain the scholarly record and will be digitally watermarked on each page as ‘Retracted’.

